# Variations in Intraplatelet Phospho-VASP Expression Due to Pre-analytical Sample Preparations, Illustration of a Quality Control Issue in Platelet Pharmacology

**Published:** 2015

**Authors:** Ahmad Gharehbaghian, Morteza Salimian, Ali Akbar Taherian, Asghar Elahi, Tahereh Khamechian, Gharib Karimi, Mehran Ghasemzadeh

**Affiliations:** a Hematology and Blood Banking Department, Shahid Beheshti University of Medical Sciences, Tehran, Iran.; b Pediatric Congenital Hematologic Disorders Research Center, Shahid Beheshti University of Medical Sciences, Tehran, Iran.; c Blood Transfusion Research Centre, High Institute for Research and Education in Transfusion Medicine, Tehran, Iran.; d Platelet Research Laboratory, Kashan University of Medical Sciences, Kashan, Iran.; e Anatomical Sciences Research Center, Kashan University of Medical Sciences, Kashan, Iran.

**Keywords:** Platelet, PRP, VASP, Pre-analytical variation, Flow cytometry, Quality control

## Abstract

Intraplatelet vasodilator-stimulated phosphoprotein (VASP) analysis is a commonly used laboratory approach for monitoring of the anti-platelet therapy with adenosine diphosphate (ADP) receptor blocking agents; however, it’s testing in clinical laboratory needs a high level of experience and proficiency. The ability to recognize how the pre-analytical variations can change the results would be helpful for the interpretation of data from intraplatelet VASP analysis. The aim of this study was to describe the possible differences of intraplatelet phospho-VASP expression between washed and platelet rich plasma (PRP) samples, both at baseline levels and following experimentally induction of VASP phosphorylation.

PRP and washed platelet samples were treated with different inducers of VASP phosphorylation, including forskolin (10 µM), prostaglandin E1 (PGE1) (50 nM) and sodium nitro-prusside (SNP) (100 µM). Untreated PRP and washed platelet samples were also included in study as baseline controls. After labeling of platelets with either anti P-Serine^157^-VASP or anti P-Serine^239^-VASP, the samples were subjected to flow cytometric analysis to monitor the levels of intraplatelet phospho-VASP expression.

Washed platelet samples tend to show increased expression of intraplatelet P-Serine^157^-VASP at baseline state and also more expression of P-Serine^157^-VASP and P-Serine^239^-VASP in response to forskolin and SNP, compared with PRP samples. Though, reduced levels of PGE1-induced VASP phosphorylation at both residues were detected for washed platelets.

In this study we have provided some background information required for performing of intraplatelet VASP analysis on differently handled platelet samples and interpretation of the obtained results.

## Introduction

Vasodilator-stimulated phosphoprotein (VASP) is a regulator of actin reorganization in platelets. VASP-actin interaction prevents capping proteins from binding to actin, therefore allows growing of the actin polymers (1). Phosphorylation of VASP at different amino acid residues such as Serine 157 (Ser157) and Serine 239 (Ser239) decreases the affinity of this molecule for binding to actin, leading to inhibition of actin reorganization in platelets (2). Platelet inhibitors such as forskolin, prostaglandin E1 (PGE1), sodium nitro-prusside (SNP) exert their effects through the stimulation of VASP phosphorylation (3).

Considering VASP as a common downstream target of various signaling pathways, an increasing attention to this molecule has been taken in platelet studies (4, 5). Likewise, in clinical laboratories, flow cytometry or enzyme linked immunosorbent assay (ELISA)-based phospho-VASP (P-VASP) analysis has been increasingly applied as an* in-vitro* approach for monitoring of anti-platelet therapy with adenosine diphosphate (ADP) receptor antagonists (6, 7). But having said that, whether applying P-VASP analysis in clinical laboratories for this purpose provides a proper degree of correlation and/or agreement with other approaches, has been a matter of serious discussions (-).

The validity of platelet experiments may be adversely influenced by inter- and intra-test variations. The procedures in pre-analytical phase of experiments are probably the most important sources of variations in platelet assessments (12, 13). The effect of washing step to induce platelet activation has been well described before (14). Even a small deviation of platelet from the physiological state of activity has a great influence on its responses to the experimental treatments (15). Considering the role of VASP phosphorylation in controlling of platelet activation, the question which may be raised is how much P-VASP dynamics in platelets can be affected by variations in pre-analytical sample preparations? No controlled study has been found that evaluated possible effects of those variations on intraplatelet P-VASP expression. The aim of this study was comparing the intraplatelet P-VASP expression between differently handled platelet samples. Therefore, to this purpose, platelet rich plasma (PRP) and washed platelet samples were subjected to comparative evaluations.

## Experimental

*Preparation of PRP and washed platelets*

According to the ethical committee guidelines of our institute, blood samples were obtained with consent from healthy donors who denied taking anti-platelet drugs for at least two weeks in plastic tubes containing sodium citrate 3.8 %. PRP was prepared from whole blood samples according to our in house protocol (16). Each PRP sample was divided into halves and washing procedure was undertaken for one part, by adapting the procedure used by others (17, 18). In order to washing, the PRP sample was diluted in citrate wash buffer (11 mM glucose, 128 mMNaCl, 4.3 mM NaH_2_PO_4_, 7.5 mM Na_2_HPO_4_, 4.8 mM sodium citrate, 2.4 mM citric acid, pH 6.5) containing freshly added 50 u/mL heparin. After centrifugation at 800 x g for 10 min at 25 °C, supernatant was removed and the pellet was rinsed without resuspension by gently adding of citrate wash buffer and removing slowly. This type of rinsing step was applied to improve recovery of platelet number at the end of the procedure. Then modified Tyrode’s-HEPES (4-(2-hydroxyethyl)-1-piperazineethanesulfonic acid) buffer containing 0.02 u/mL apyrase was added on platelet pellet. The sample was then incubated for 15 min at 37 °C before resuspension. The concentration of platelets in washed samples was adjusted to the equal level of PRP samples and allowed to rest at least for 1 hour at 25 °C prior to be used in experiments.

*Experimental treatments on washed and PRP samples*

PRP and washed platelet samples were incubated in the presence of 50 nM PGE1 (Sigma), 10 µM forskolin (Calbiochem), or 100 µM SNP (Merck). Untreated PRP and washed platelet samples were also included in each run of the experiments as baseline controls. After 10 min incubation at room temperature on shaker, the treatments were terminated by adding Para-formaldehyde (1% final concentration).

*Flow cytometry analysis*

The platelet samples which were fixed in Para-formaldehyde, permeabilized in 0.5% Triton X-100 in PBS for 30 min at 4 °C. Samples containing 20x10^6 ^platelets were then incubated with a specific primary antibody (1/200 in 2% bovine serum albumin, BSA, in PBS) for 14 hour at 4 °C with gentle shaking. Primary antibodies utilized were polyclonal rabbit anti-P-Ser^157^-VASP and polyclonal rabbit anti-P-Ser^239^-VASP (from Cell Signaling). After a washing step, FITC-conjugated goat anti-rabbit polyclonal IgG (1/500, Santa Cruz Biotechnology) was used as secondary antibody. Flow cytometric analysis of 10,000 events in platelet area / sample was performed using a flow cytometer (CyFlow®Space, Partec GmbH, Germany). Platelets were gated by forward (FSC)/ side (SSC) scatter characteristics, the median fluorescence intensity (MFI) was quantified for gated events using Flomax instrument software. In each run of experiments, a platelet sample labeled only with secondary antibody was included as negative control to exclude the fluorescence due to non-specific binding of secondary antibodies.

After each run of experiments on either PRP or washed platelet samples, MFI value obtained for untreated sample was considered as baseline and the percent of MFI shift from baseline, representing the percent of induced change in P-VASP expression, for a forskolin-, PGE1-, or SNP-treated sample was calculated, using formula

*Statistical analysis*

Statistical significance was analyzed using non-parametric analysis of variance.

## Results and Discussion

**Figure1:**
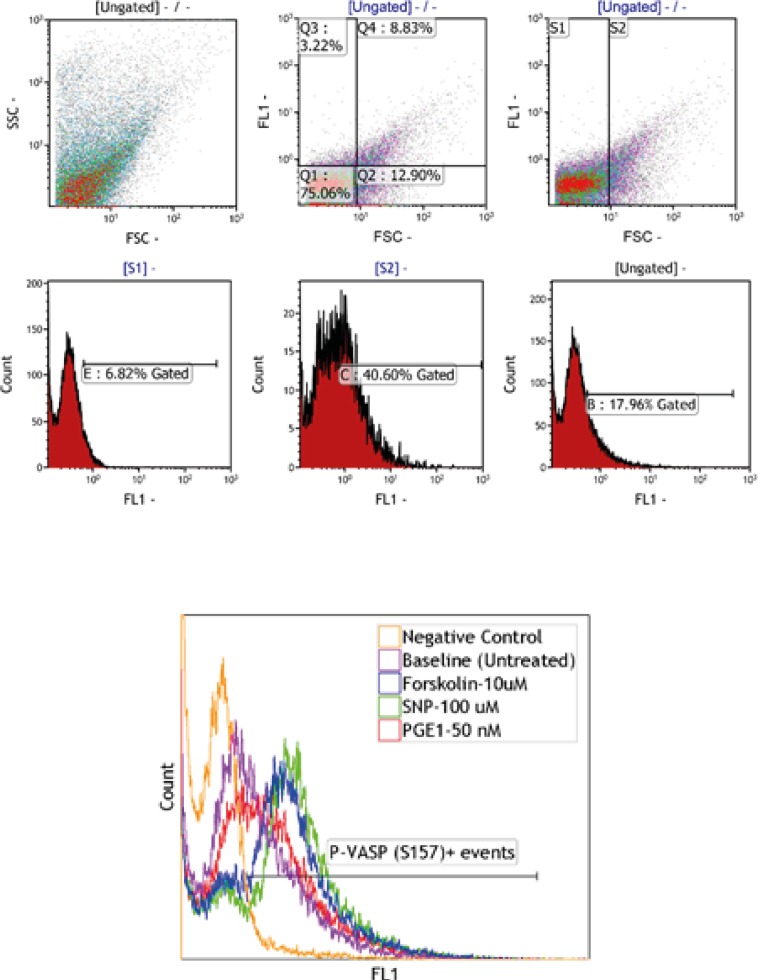
A) Washed platelets treated with either anti-P-ser^157^-VASP or anti-P-ser^239^-VASP antibodies in the presence of PGE1 (50 nM), were labeled by FITC conjugated secondary antibody. To analyze VASP phosphorylation, platelets were then subjected to flow cytometry. **Platelets were detected by FSC/S**SC characteristics. **FL1-FSC** dot plots **demonstrate two distinct platelet populations (S1 and S2) which then gated separately to be analyzed in** **FL1** histograms. B) The overlaying histograms display the levels of VASP phosphorylation in platelets treated with different agonists including; forskolin (10 µM), SNP (100 µM) and PGE1 (50 nM). The histograms represented the whole population of platelets in **FL1-FSC** dot plots (S1+S2).

**Figure 2:**
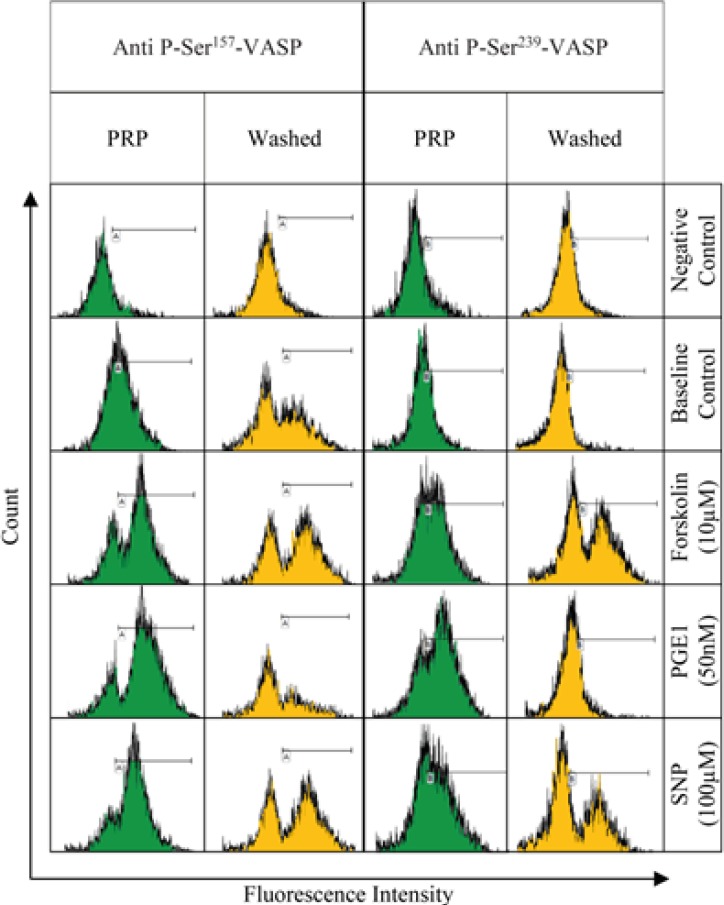
The histograms have been generated from flow cytometry analysis on platelets which were labeled with either anti-P-ser^157^-VASP or anti-P-ser^239^-VASP as primary antibodies and then FITC-conjugated secondary antibody. The platelet samples labeled only with secondary antibody have been used as negative control. The histograms represented the S2 population of platelets in **FL1-FSC** dot plots. Comparing the histograms obtained for PRP and washed samples suggests some variation in the levels of intraplatelet P-VASP expression at baseline (control) and/or in response to forskolin (10 µM), PGE1 (50 nM), or SNP (100 µM).

PRP and washed platelet samples, labeled by anti-P-ser^157^-VASP and anti-P-ser^239^-VASP, were subjected to flow cytometric analysis. The gating strategy which was applied has been described in Figure 1. Fluorescence intensity histograms from one representative experiment out of six performed is shown in Figure 2. The histograms obtained from conducting the P-VASP analysis on PRP and washed platelet, indicates different behavior of PRP and washed platelets to express intracellular P-VASP at baseline state and/or in response to different inducers of VASP phosphorylation. 

**Figure 3:**
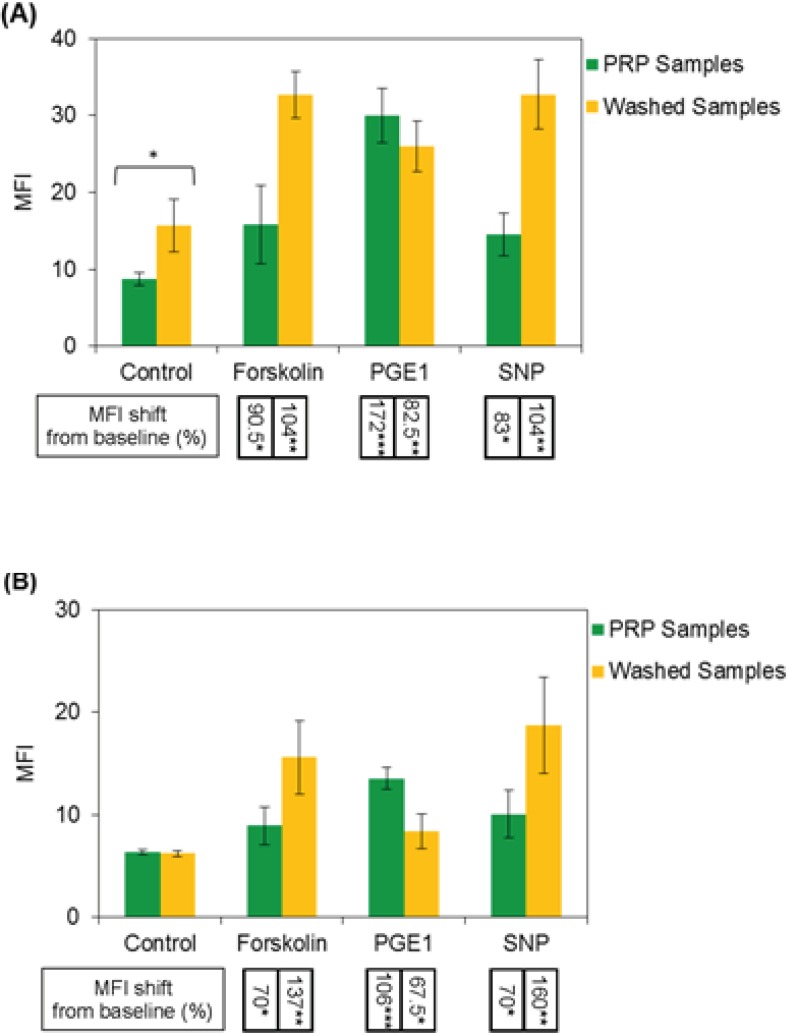
Median fluorescence intensity (MFI) values indicating the levels of intraplatelet P-Ser^157^-VASP and P-Ser^239^-VASP have been presented in graphs (A) and (B), respectively. The average percent values of MFI shifts from baseline (control), induced by forskolin, PGE1 and SNP, have been presented below the associated columns. From the results on graph (A), significant increase in baseline expression of P-Ser^157^-VASP in washed platelets could be noted; however, the results presented in graph (B) indicate no significant differences in baseline expression of P-ser^239^-VASP between washed and PRP platelets. As can be seen from the data in graphs, washed platelet samples have shown more levels of intraplatelet P-Ser^157^-VASP and P-Ser^239^-VASP induction in response to forskolin and SNP, in comparison with PRP samples; however these samples have expressed reduced levels of P-VASP at both residues in response to PGE1. Data shown are from six runs of experiments using platelet samples obtained from six different donors (n=6).

Expressions of intraplatelet P-ser^157^-VASP and P-ser^239^-VASP were quantified by flow cytometry software, as median fluorescence intensity value (MFI). From the graphs in Figure 3, the MFI values obtained for PRP and washed platelet samples could be compared. The results, as shown in graph (A), indicate more expression of P-Ser^157^-VASP at baseline level for washed platelets, however, no significant differences were found between two types of the samples in expression of P-Ser^239^-VASP at their baseline levels, graph (B). The graphs also indicate more levels of forskolin- and SNP-induced VASP phosphorylation at Serine 157, 239 for washed platelets, whereas, reduced levels of PGE1-induced VASP phosphorylation at both residues were detected for washed platelets.

Research and clinical laboratory experiments on platelets may be conducted on whole blood, PRP, or washed platelet samples(19). Variation in pre-analytical processing of samples may cause considerable morphological and functional changes in platelets. To avoid such variations and also save time and labor, clinical laboratories prefer to conduct platelet experiments on less handled samples. Likewise, analyzing of intraplatelet VASP phosphorylation on whole blood samples using ELISA- or flow cytometry-based standardized kits has been considered by clinical laboratories. In spite of this, several possible variations such as those associated with blood sampling remain to be controlled (20). Therefore despite using the kits, performing of intraplatelet P-VASP analysis in a clinical laboratory still needs a high level of experience and proficiency. On the other hand, washed platelets may be preferably chosen to be applied in research studies, to ensure that the effects of matrix variables on obtained results are excluded. We hope our findings provide a better understanding about the changes, that might be occur in intraplatelet P-VASP expression due to variation in pre-analytical sample preparation. Considering a proper approach for dealing with those changes may help improving of validity and reproducibility of P-VASP measurements, both in clinical and research investigations.

The present study was designed to evaluate intraplatelet VASP phosphorylation by flow cytometry method. Specific interactions of applied antibodies in flow cytometric analysis of phosphorylated VASP had been confirmed by western blot analysis, before using in experiments (data not shown). Our results indicated more levels of P-Ser^157^-VASP expression at baseline for washed platelets, compared with the platelets existed in PRP samples. The elevation of Ser^157^-VASP expression in thrombin and collagen activated platelets has been also reported, previously (21, 22). Platelets are highly susceptible to be priming along with *in-vitro* manipulations. Such an artifactual pre-activation of platelets can be started from the time of blood sampling and progress with further manipulations, but under controlled condition this status is usually reversible and platelets tend to return to their resting phenotype again (23). In this study high attention was taken to prevent the progress of platelet activation during washing procedure and while platelet endured some reversible shape changes after washing steps, the monitoring of platelets after washing procedure revealed no significant increases in P-selectin expression.

The results from this study showed no significant differences in the levels of P-Ser^239^-VASP expression at baseline state between washed and PRP samples. This finding is consistent with the data from previous studies, indicating unchanged levels of P-Ser^239^-VASP in agonist activated platelets (21).

Although Ser157 residue on VASP molecule is the primary target of phosphorylation by PGE1, commonly used flow cytometry kits available for monitoring of anti-P2Y12 drugs, instead evaluate the levels of P-Ser^239^-VASP expression. It seems reasonable approach; because according to our findings, variation in the pre-analytical sample preparations may cause fewer changes in baseline expression of P-Ser^239^-VASP in platelets, compared with those of Ser157 residue, graph B in Figure 2.

After treatment of washed and PRP samples in the presence of different P-VASP inducers, washed platelets revealed more levels of forskolin- and SNP-induced VASP phosphorylation but less extents of PGE1-induced P-VASP expression, compared with the platelets in PRP samples. Forskolin and PGE1 are known to stimulate intraplatelet VASP phosphorylation by similar mechanism, which is inducing of cAMP (cyclic Adenosine Monophosphate) cascade (24). In spite of this, washing procedure was able to modulate their effects in opposite directions; this might be explained by releasing of some contents of endogenous ADP from manipulated platelets in experimental environment. It may worth mentioning that susceptibility of PGE1-mediated adenylate cyclase activation to be reversed in the presence of ADP has been established before (25, 26).

Variability of intraplatelet P-VASP expression observed between PRP and washed platelet samples might be related to the mechanical stress, which could be exerted on platelets by centrifugation and other procedures of washing step, however, possible effects of matrix components within different group of samples cannot be also ignored. One other caveat that must be noted here is that the results from this study may have been influenced by test-specific properties of applied method (flow cytometry). Further investigations using ELISA-based P-VASP analysis are required to be undertaken for estimating the effect of those factors.

## Conclusion

Our results suggested that the dynamics of phospho-VASP expression in platelets is sensitive to be changed due to variation in pre-analysis samples preparations. The Patterns of observed changes were different, regarding the type of P-VASP inducer and also the amino acid residue of phosphorylation on VASP molecule. The data provided from this study might be considered as an integral part of background information that is required for performing of intraplatelet P-VASP analysis and interpretation of the results. An extensive attention to avoid platelet stimulation during blood sampling and processing might be important to prevent artifactual variations of P-VASP data in platelet pharmacology.
